# Designing a reliable machine learning system for accurately estimating the ultimate condition of FRP-confined concrete

**DOI:** 10.1038/s41598-024-69990-4

**Published:** 2024-09-03

**Authors:** Meysam Alizamir, Aliakbar Gholampour, Sungwon Kim, Behrooz Keshtegar, Woo-tai Jung

**Affiliations:** 1 Institute of Research and Development, Duy Tan University, Da Nang, Vietnam; 2 School of Engineering & Technology, Duy Tan University, Da Nang, Vietnam; 3 College of Science and Engineering, Tonsley, Flinders University, Adelaide, SA 5042 Australia; 4 Department of Railroad Construction and Safety Engineering, Dongyang University, Yeongju, 36040 Republic of Korea; 5 Civil Engineering Department, University of Zabol, Zabol, Iran; 6 Research Fellow. Department of Structural Engineering Research, Korea Institute of Civil Engineering and Building Technology, Gyeonggi, Republic of Korea; 7 Key Laboratory of Modelling and Simulation-based Reliability and Optimization, University of Zabol, Zabol, Iran

**Keywords:** Fiber-reinforced polymer, Machine learning, Empirical model, Ultimate condition, Compressive strength, Concrete confinement, Civil engineering, Computer science

## Abstract

Precisely forecasting how concrete reinforced with fiber-reinforced polymers (FRP) responds under compression is essential for fine-tuning structural designs, ensuring constructions fulfill safety criteria, avoiding overdesigning, and consequently minimizing material expenses and environmental impact. Therefore, this study explores the viability of gradient boosting regression tree (GBRT), random forest (RF), artificial neural network-multilayer perceptron (ANNMLP) and artificial neural network-radial basis function (ANNRBF) in predicting the compressive behavior of fiber-reinforced polymer (FRP)-confined concrete at ultimate. The accuracy of the proposed machine learning approaches was evaluated by comparing them with several empirical models concerning three different measures, including root mean square errors (RMSE), mean absolute errors (MAE), and determination coefficient (R^2^). In this study, the evaluations were conducted using a substantial collection of axial compression test data involving 765 circular specimens of FRP-confined concrete assembled from published sources. The results indicate that the proposed GBRT algorithm considerably enhances the performance of machine learning models and empirical approaches for predicting strength ratio of confinement (*f′*_*cc*_*/f′*_*co*_) by an average improvement in RMSE as 17.3%, 0.65%, 66.81%, 46.12%, 46.31%, 46.87% and 69.94% compared to RF, ANNMLP, ANNRBF, and four applied empirical models, respectively. It is also found that the proposed ANNMLP algorithm exhibits notable superiority compared to other models in terms of reducing RMSE values as 9.67%, 11.29%, 75.11%, 68.83%, 73.64%, 69.49% and 83.74% compared to GBRT, RF, ANNRBF and four applied empirical models for predicting strain ratio of confinement (ε_*cc*_/ε_*co*_), respectively. The superior performance of the GBRT and ANNMLP compared to other methods in predicting the strength and strain ratio confinements is important in evaluating structural integrity, guaranteeing secure functionality, and streamlining engineering plans for effective utilization of FRP confinement in building projects.

## Introduction

The integration of machine learning (ML) into the civil engineering field has introduced new era of predictive accuracy and efficiency^1^. Among its many applications, the prediction of ultimate condition of fiber-reinforced polymer (FRP)-confined concrete stands out as a remarkable example of how ML methods are reshaping the field^2^. The FRP wrapping of concrete has been one of the most broadly applied techniques to retrofit current concrete elements^3^. This technique is basically used to enhance the concrete strength and ductility through providing confinement mechanism to the lateral dilation of concrete^4^. The structural members manufactured with FRP-confined concrete composite have complex responses to external loading under different uncertainties as design variables^5^. Therefore, to design the structural members made with the composite accurately, a simple and efficient model is required for estimating the FRP-confined concrete performance^6,6^.

Predicting the ultimate behavior of FRP-confined concrete under axial compression, including compressive strength and ultimate axial strain, is a complex job because of the intricate interplay of different factors, including concrete and confinement properties^8^. Several studies have been conducted for predicting the compressive behavior of FRP-confined concrete by developing analytical models^6,7, 9–15^. Nevertheless, the accuracy of the models has been questionable because they used a database with either relatively small number of test results or input variables. They also lack the capacity to capture the multifaceted relationships within the data. ML strategies have appeared as a powerful approach for estimating the ultimate behavior of FRP-confined concrete^16–22^. However, they were not able to find the impact of all crucial factors, they were computationally complicated models, or they were not efficient to handle large size database. Therefore, more accurate and efficient techniques are required for estimating the compressive behavior of the FRP-confined concrete at ultimate.

Owing to its nonlinear mapping capability and simplicity, artificial neural network (ANN) has been the most extensively utilized approach for estimating the behavior of FRP-confined concrete^23^. However, the training process is one of the critical issues to provide the robust, accurate and efficient design relations using ANN^24^. Therefore, the multilayer perceptron (MLP) method was applied to train the multilayer neural network model^25^. The model of ANN coupled with MLP (ANNMLP) was utilized for predicting the compressive and tensile strengths of concretes^26–29^, the dynamic response of buildings^30^, the surface chloride concentration of concrete^31^^,^ the load carrying capacity of glass FRP-reinforced concrete^32^, and the bond strength between reinforcing steel rebar and concrete^33,34^. The ANNMLP has had advantages of modeling complex nonlinear relationships in data and automatically acquiring pertinent attributes from the input data^35^. The ANN was also coupled with radial basis function (RBF) as activation functions in the hidden layers of ANN to develop a model capable of approximating complex functions with nonlinearity and solving various types of ML tasks^36^. The model of ANN coupled with RBF (ANNRBF) was utilized for estimating the compressive and tensile strengths of concretes^37–40^, the recycled aggregate concrete’s elastic modulus^41^, the axial compression capacity of steel tubular concrete columns with square cross-section^42^, and the load carrying capacity of glass FRP-reinforced concrete^32^.

Gradient boosted regression tree (GBRT) is another a ML method which combines forecasting capabilities of numerous weak learners (typically decision trees) to formulate a stronger forecasting tool^43^. The GBRT model was utilized for predicting the compressive and tensile strengths of concretes^44,45^, the shear strength of steel fiber-reinforced concrete beams^46^, the carbonation depth of recycled aggregate concrete^47^, the surface chloride concentration of concrete^31^, the bond strength of FRP-concrete^48^, the load carrying capacity of steel tubular concrete columns with circular cross-section^49,50^, the corroded reinforced concrete beams’ shear strength^51^, the shear strength of reinforced concrete deep beams^52^, and the bond strength between concrete and profiled steel^53^. The benefits of GBRT have been the highly predicting power, handling different data types and capturing nonlinear patterns in the data due to the hierarchical structure of decision trees^1,43^. Random forest (RF) is another popular ensemble learning algorithm, and like GBRT, it belongs to the family of decision tree-based algorithms^54^. This model was applied for predicting the compressive and tensile strengths of concretes^44,45, 55, 56^, the flexural strength^57^ and shear strength^58–60^ of steel fiber-reinforced concrete beams, the shear strength of joints of reinforced concrete columns and beams^61^, the surface chloride concentration of concrete^31^, the crack depth of reinforced concrete^62^, the load carrying capacity of steel tubular concrete columns with circular cross-section^50^, the flexural strength of concrete beams strengthened with FRP^60^, and the bond strength of profiled steel–concrete in steel-reinforced concrete composites^53^. The advantages of the RF have been the reduced overfitting, robustness, automatic feature selection, and efficiency in training multiple trees in parallel^54^. Based on the results obtained from existing studies, the advanced models of ANNMLP, ANNRBF, GBRT and RF offer enhanced capacity to handle complex relationships, nonlinearity, and high-dimensional data in comparison to other ML models. They excel in capturing subtle patterns, making accurate predictions, and addressing challenges like overfitting and feature importance.

Based on the literature review, there is no study to date in applying ANNMLP, ANNRBF, GBRT and RF for accurate predicting of the FRP-confined concrete’s ultimate condition. To address this research gap, in this study these models are used for estimating the compressive strength and ultimate axial strain of concrete composite cores confined with the FRP jackets. A summary of the experimental database is initially presented, and the models’ description is then presented. The most accurate existing empirical models for predicting the ultimate points of FRP-confined concrete are also presented and the prediction performances of the ML and the existing empirical models are finally evaluated.

### Research significance

Predicting the compressive behavior of FRP-confined concrete is significant due to its implications in enhancing the performance and efficiency of concrete structures, such as building construction, bridges, marine structures, and seismic retrofitting. The FRP materials, such as carbon and glass FRPs, are increasingly used to confine concrete because they improve the concrete’s strength, ductility, and durability under compressive loads. The accurate prediction of the compressive behavior of the FRP-confined concrete is crucial for several reasons including optimizing design of concrete structures and ensuring that the structures meet safety standards and performance requirements without overdesigning, thereby reducing materials cost and environmental impact.

The existing ML models used to predict the compressive behavior of FRP-confined concretes were not able to find the impact of all crucial factors, they were computationally complicated models, or they were not efficient to handle large size database. Therefore, more accurate and efficient techniques are required for estimating the compressive behavior of the FRP-confined concrete at ultimate. The GBRT, RF, ANNMLP and ANNRBF were chosen in this study because: the robustness to outliers, interpretability, and scalability of the GBRT make it a powerful tool to handle non-linear relationships and capture interactions between variables^1^, the RF offers reduced overfitting, robustness, automatic feature selection, and efficiency in training multiple trees in parallel^54^, the ANNMLP has advantages of modeling complex nonlinear relationships in data and automatically acquiring pertinent attributes from the input data^35^, and the RBF in the AANRBF acts as an activation function in the hidden layers of the ANN to model complex functions with nonlinearity and solve various types of ML tasks^36^.

## Experimental database

This study gathered data for circular concrete specimens confined with FRP, featuring a ratio of height to diameter that is below 3, sourced from available literature for calibrating the proposed models for predicting strength ratio of confinement (*f′*_*cc*_*/f′*_*co*_) and strain ratio of confinement (*ε*_*cc*_/*ε*_*co*_) parameters. Therefore, a substantial experimental dataset was generated, encompassing the outcomes of 765 cylindrical concrete specimens that were reinforced with unidirectional fibers, specifically oriented in the hoop direction. The study drew upon data from multiple references, establishing a comprehensive and diverse dataset that bolsters the robustness of analysis of the research^6,7, 10, 63–69^. The effectiveness of the suggested methods was assessed through comparison with multiple empirical formulations^6^^,^^7^^,^^10^^,^^11^^,^^12^^,^^15^. Three distinct performance indicators were employed in this evaluation: the root mean square deviation (RMSE), the mean absolute deviation (MAE), and the coefficient of determination (R^2^). Moreover, in this study, 80 percent of the dataset was used for training, and the remaining 20 percent was applied for testing. The statistical properties of both training and testing database are presented in Table 1. The table provides details pertaining to each individual specimen, including the total thickness (*t*) of the fibers, diameter (*D*) of the concrete, strength of unconfined concrete (*f′*_*co*_), strength of confined concrete under compression (*f′*_*cc*_), axial strain (*ε*_*co*_) associated with the unconfined concrete strength, maximum axial strain (*ε*_*cc*_) of confined concrete at failure, modulus of elasticity (*E*_*f*_) of the fiber, strength in tension (*f*_*fu*_) of the fiber, strain at which hoop rupture occurs in the FRP material (*ε*_*h,rup*_), ratio of strain (*ρ*_ε_), ratio of confinement stiffness (*ρ*_*K*_) and the ratio of pressure exerted in the lateral direction for confinement (*f*_*l*_*/f′*_*co*_). In the dataset, carbon FRP (CFRP) was used to confine 491 specimens, glass FRP (GFRP) was used to confine 159 specimens, and aramid FRP (AFRP) was used to confine 115 specimens.

**Table 1:** Statistical properties of variables employed in this research.

	Phase	Variable	Unit	Avr	Min	Max	St. Dev	Skewness
Dataset	Training	D	mm	152.89	51	406	47.43	2.71
		t	mm	0.82	0.08	7.26	0.90	2.45
		f′_co_	MPa	52.35	16.60	188.20	30.39	1.79
		f′_cc_	MPa	96.06	24.10	372.20	48.18	1.34
		ε_co_	%	0.24	0.18	0.34	0.03	0.89
		ε_cc_	%	1.86	0.23	6.20	1.03	0.96
		ε_h,rup_	%	1.20	0.10	4.98	0.57	0.99
		E_f_	GPa	181.50	13.60	640	116.38	0.84
		f_fu_	MPa	2754.9	230	4441	1326.5	− 0.43
		ρ_K_		0.06	0.008	0.68	0.06	3.69
		f_l_/f′_co_	–	0.29	0.005	2.07	0.25	3.13
		ρ_ε_		4.99	0.28	23.03	2.49	1.19
		f′_cc_/f′_co_	–	1.99	0.83	6.83	0.82	2.25
		ε_cc_/ε_co_	–	7.82	0.86	27.99	4.72	1.27
	Testing	D	mm	159.85	70	406	48.01	3.74
		T	mm	0.80	0.10	5.84	0.95	2.89
		f′_co_	MPa	49.23	20.57	127.10	24.60	1.43
		f′_cc_	MPa	89.98	32.90	201.90	43.56	1.07
		ε_co_	%	0.24	0.19	0.31	0.02	0.78
		ε_cc_	%	1.88	0.32	4.84	1.01	0.68
		ε_h,rup_	%	1.26	0.19	3.13	0.58	0.63
		E_f_	GPa	175.23	10.50	662.50	120.73	0.93
		f_fu_	MPa	2643.5	220	4441	1312.4	− 0.37
		ρ_K_		0.05	0.007	0.22	0.03	1.91
		f_l_/f′_co_	**–**	0.27	0.01	1.27	0.19	1.98
		ρ_ε_		5.28	0.74	13.35	2.51	0.65
		f′_cc_/f′_co_	**–**	1.92	0.97	4.29	0.67	1.46
		ε_cc_/ε_co_	**–**	7.88	1.24	21.62	4.47	0.88

In the application range of empirical models, diverse mathematical models of Keshtegar et al.^6,7^ were implemented by Mander et al.^70^, Teng et al.^13^, and Wang and Wu^71^. Also, the chaos control algorithm (CCA) calibrated the unknown coefficient of nonlinear equations of^6^. The proposed equation of Ozbakkaloglu and Lim^10^ was developed utilizing nonlinear form of confinement stiffness and lateral confining pressure ratio (*f*_*l*_*/f′*_*co*_). The mathematical form of Wu and Wei^15^ employed a power function and calculated the final strength by applying the nonlinear framework of lateral confining pressure ratio (*f*_*l*_*/f′*_*co*_). The nonlinear pattern of Sadeghian and Fam^12^ separated the ratio of confinement stiffness and ratio of strain.

## The process of confining with FRP

The procedure of utilizing FRP for confinement entails enveloping concrete structures with materials like carbon or glass fibers^6,7^. This process improves some properties of the concrete, such as the ductility, strength, and load-bearing capability, offering increased resilience against deformation, cracking, and failure when subjected to diverse applied loads^6,7^. The ultimate strength and strain of FRP-confined concrete can be significantly improved by the effective lateral confining pressure. Based on the lateral confining pressure, most of the current approaches that estimate the ultimate values of strength and strain of cylindrical FRP-confined concrete specimens were developed^2^. Assuming a uniform distribution, the lateral confinement is considered to surround the circumference of the concrete sections with a circular shape^6,7^. The equation provided below is utilized to calculate the actual ultimate confining pressure (*f*_*l*_) of the FRP jacket at the point of rupture.

1 $${f}_{l}=\frac{2{E}_{f}{t}_{f}}{D}{\varepsilon }_{h.rup}$$

During axial compression, the lateral expansion of concrete gives rise to the principal stress parallel to the cross-sectional plane, known as the lateral confining pressure^2^. The determination of parameter of the lateral confining pressure can be achieved by utilizing the following relationship involving the confinement stiffness ratio (*ρ*_*K*_) and the strain ratio (*ρ*_ε_)^2^.

2 $$\frac{{f}_{l}}{{f}_{co}^{\prime}}={\rho }_{\text{K}} {\rho }_{\upvarepsilon }$$

The values of *ρ*_K_ and *ρ*_ε_ are obtained through a specific calculation as follows^2^:

3 $$\rho _{{\text{K}}} = \frac{{2E_{f} t_{f} }}{{D\left( {{\raise0.7ex\hbox{${f_{{co}}^{{\prime }} }$} \!\mathord{\left/ {\vphantom {{f_{{co}}^{{\prime }} } {\varepsilon _{{co}} }}}\right.\kern-\nulldelimiterspace} \!\lower0.7ex\hbox{${\varepsilon _{{co}} }$}}} \right)}}$$

4 $${\rho }_{\upvarepsilon }=\frac{{\varepsilon }_{h.rup}}{{\varepsilon }_{co}}$$

where *f′*_*co*_ represents the compressive strength of the concrete when no confining pressure is applied. *ε*_*co*_ denotes the axial strain of the concrete corresponding to *f′*_*co*_, that can be calculated via the following equation^6,7^:

5 $${\varepsilon }_{co}=\frac{{f}_{co}^{{\prime}0.225}}{1000}{\left(\frac{152}{D}\right)}^{0.1}{\left(\frac{2D}{H}\right)}^{0.13}$$

where *H* and *D* represent the height and diameter of the specimen, respectively, measured in millimeters. The effectiveness of the machine learning methods suggested was assessed through a comparison with various empirical models outlined in Tables 2 and 3 for both *f′*_*cc*_*/f′*_*co*_ and ε_cc_/ε_co_.

**Table 2:** Empirical approaches for compressive strength of FRP-confined concrete (*f*_*cc*_*/f*_*co*_) prediction.

Model	Expression
Keshtegar et al.^6,7^	(*f′*_*cc*_*/f′*_*co*_) = $1 + \left( {3.23 + 4.8\rho_{K}^{2.5} } \right)\left( {\frac{{f_{l} }}{{\mathop f\limits^{\prime }_{co} }}} \right)^{0.95}$.
Ozbakkaloglu and Lim^10^	(*f′*_*cc*_*/f′*_*co*_) = $1 + 0.0058\frac{{K_{l} }}{{\mathop f\limits^{\prime }_{co} }} + 3.22\left( {\frac{{f_{l} }}{{\mathop f\limits^{\prime }_{co} }} - \frac{{f_{lo} }}{{\mathop f\limits^{\prime }_{co} }}} \right)$ ${K}_{l}=2{E}_{f}{t}_{f}/D$ $f_{lo} = K_{l} \left( {0.43 + 0.009\frac{K}{{\mathop f\limits^{\prime }_{co} }}} \right)\varepsilon_{co}$
Sadeghian and Fam^12^	(*f′*_*cc*_*/f′*_*co*_) = $1+(2.77{\rho }_{K}^{0.77}-0.07){\rho }_{\varepsilon }^{0.77}$
Pham and Hadi^11^	(*f′*_*cc*_*/f′*_*co*_) = $0.75 + 1.8\frac{{f_{l} }}{{\mathop f\limits^{\prime }_{co} }} + 5.7\frac{{t_{f} }}{{D\mathop f\limits^{\prime }_{co} }} + \frac{13}{{\mathop f\limits^{\prime }_{co} }}$

**Table 3:** Empirical approaches for compressive strain of FRP-confined concrete (*ε*_*cc*_/*ε*_*co*_) prediction.

Model	Expression
Keshtegar et al.^6,7^	(*ε*_*cc*_/*ε*_*co*_) = $1 + \left( {7.31 + 2.06\rho_{{\upvarepsilon }}^{0.8} } \right)\left( {\frac{{f_{l} }}{{\mathop f\limits{\prime}_{co} }}} \right)^{0.6}$.
Ozbakkaloglu and Lim^10^	(*ε*_*cc*_/*ε*_*co*_) = $2 - \frac{{\mathop f\limits{\prime}_{co} - 20}}{100} + 0.271\left( {\frac{{K_{i} }}{{\mathop f\limits{\prime}_{co} }}} \right)^{0.9} \frac{{\varepsilon_{h.rup}^{1.35} }}{{\mathop \varepsilon \limits{\prime}_{co} }}$ $K_{l} = 2E_{f} t_{f} /D$
Sadeghian and Fam^12^	(*ε*_*cc*_/*ε*_*co*_) = $1.5+6.78{\rho }_{K}^{0.63}{\rho }_{\varepsilon }^{1.08}$
Wu and Wei^15^	(*ε*_*cc*_/*ε*_*co*_) = $1.75 + 140\frac{{f_{l} }}{{\mathop f\limits{\prime}_{co} }}\varepsilon_{h.rup}^{0.6}$

## Methods

**Figure 1:**
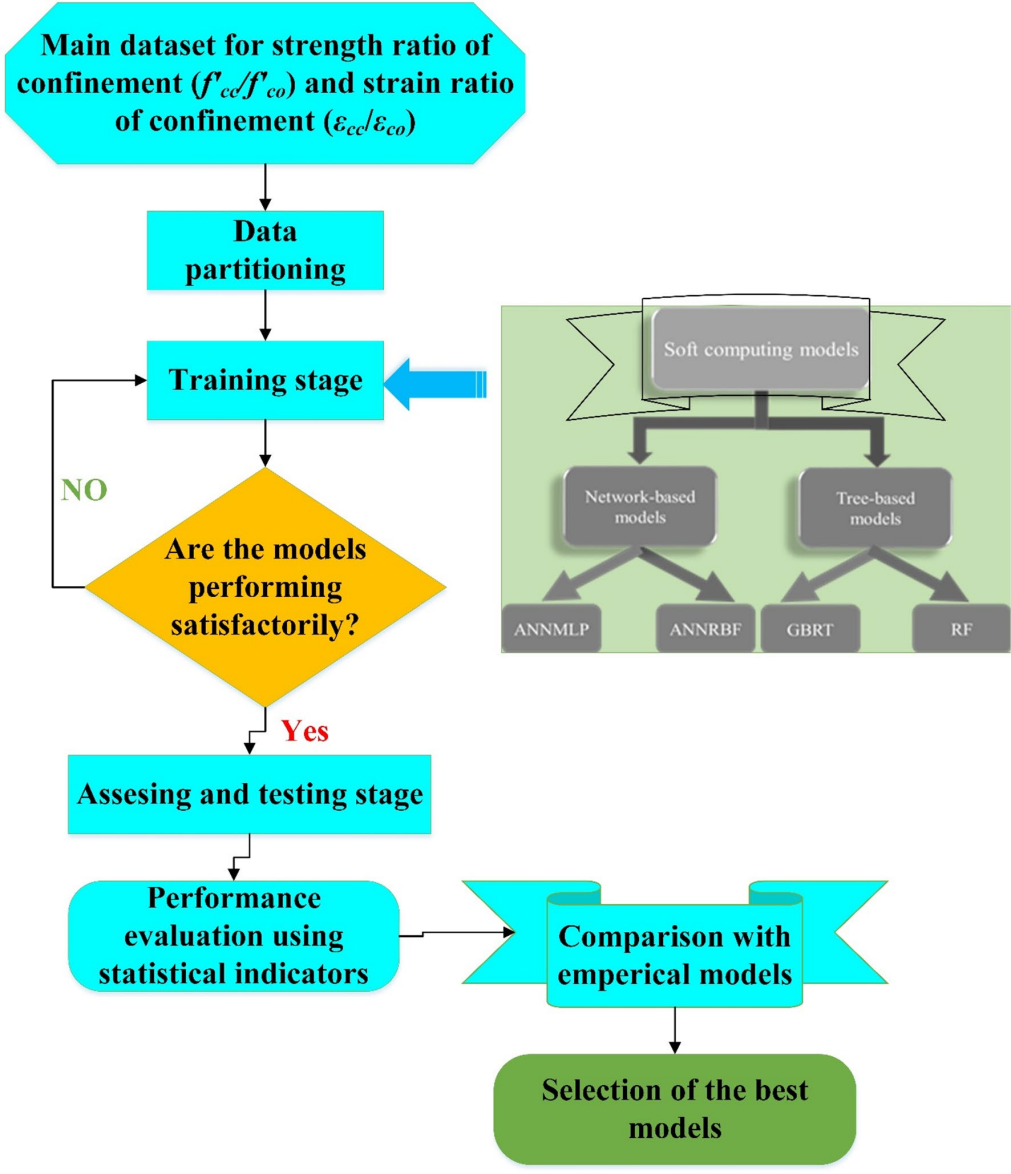
Flowchart of study methodology.

In this study, four different ML models were applied for estimating the ultimate strength and strain of FRP‑confined concrete using two different categories, including tree-based and network-based models. The first category included GBRT and RF and the second category consisted of ANNMLP and ANNRBF models. For finding the most reliable model in modelling the strength and strain of the FRP‑confined concrete, results of the tree-based and network-based models were compared with four different empirical models. Figure 1 shows diagram of the used models in this study.

### Gradient boosting regression tree (GBRT)

Gradient boosting tree is one of the robust and versatile ensemble techniques that can be used for both regression (GBRT) and decision (GBDT) problems^72^. The GBRT establishes a model by aggregating different classes of weak patterns or learners (decision trees) in an iterative boosting procedure. This method can be applied to make a class prediction by generating a group of decision trees by using the greedy way to obtain more accurate outputs during training phase^73^^,^^74^.

For a given training data, $(x,y) = \left\{ {(x_{i} ,y_{i} )} \right\}_{i = 1}^{n}$, where x and y are input and output parameters, respectively. By considering *J* as the number of leaves in per tree, the regression tree (RT) can be defined as follows^72^:

6 $$g_{m} (x) = \sum\limits_{j = 1}^{J} {b_{jm} I(x \in R_{jm} )}$$

where *R* and *m* denote number of sub class of input parameters and index for tree that splits independent variable, respectively. In each iteration, the gradient descent (GD) minimizes the difference between real and modelled data to renew the equation at each step, which can be represented as follows^72^^,^^74^:

7 $$f_{m} (x) = f_{m - 1} (x) + \rho_{m} g_{m} (x)$$

8 $$\rho_{m} = \arg \min_{\rho } \sum\limits_{i = 1}^{n} {L(y_{i} ,f_{m - 1} (x_{i} ) + \rho g_{m} (x_{i} ))}$$

Finally, the upgraded model is expressible as follows:

9 $$f_{m} (x) = f_{m - 1} (x) + \sum\limits_{j = 1}^{J} {\rho_{jm} I(x \in R_{jm} )}$$

10 $$\rho_{m} = \arg \min_{\rho } \sum\limits_{{x \in R_{jm} }}^{n} {L(y_{i} ,f_{m - 1} (x_{i} ) + \sum\limits_{j = 1}^{J} {\rho I(x \in R_{jm} ))} }$$

**Figure 2:**
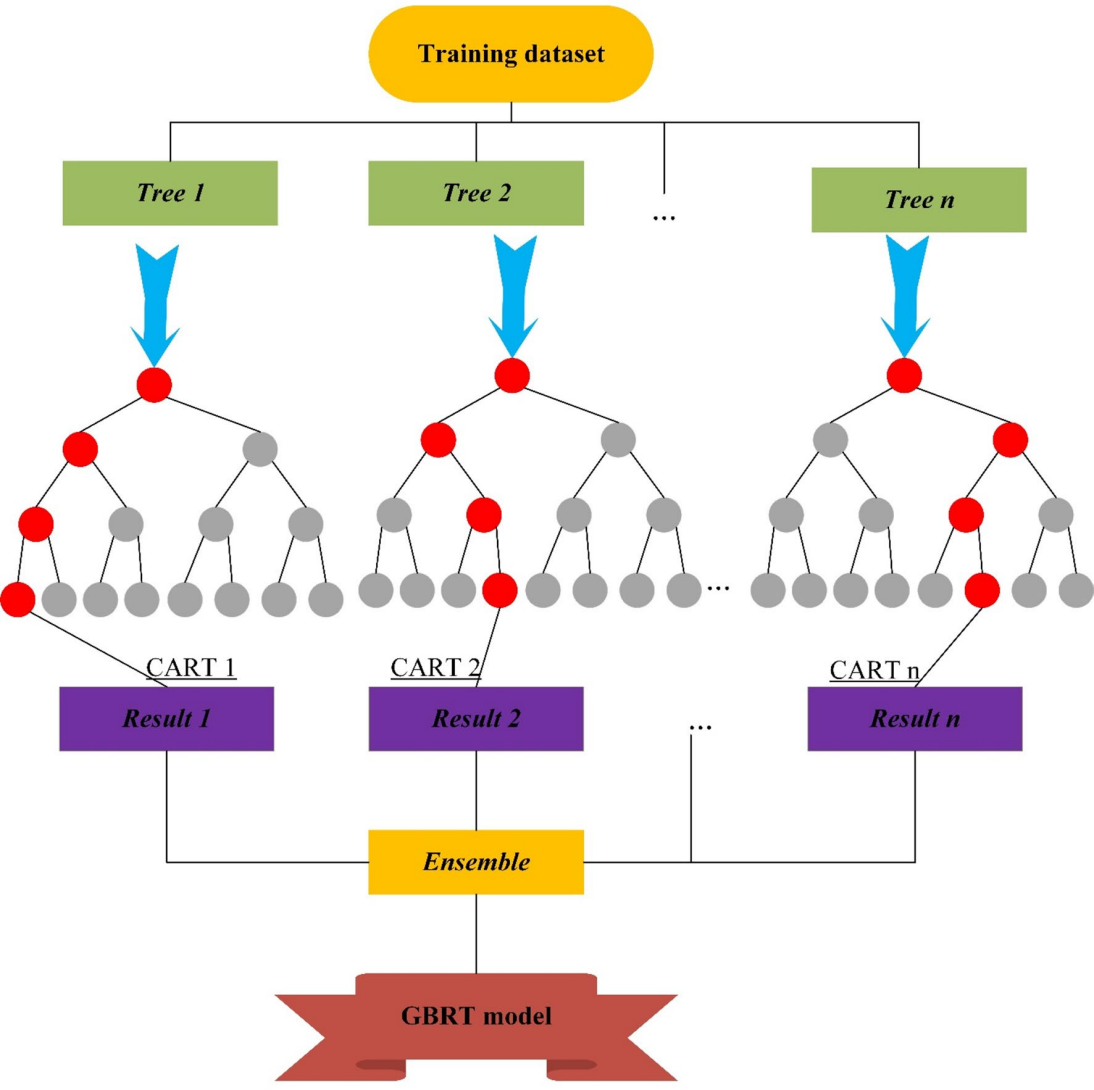
The typical structure of GBRT model.

The most important feature of the GBRT is its computational efficiency and effectively avoiding over-fitting problems by tuning some parameters like learning rate and number of basic trees in each iteration. It should be also mentioned that the shrinkage coefficient controls over-fitting in this model. Figure 2 shows schematic structure of GBRT model.

### Random forest (RF)

The RF was suggested by Breiman^75^. The main idea of the RF is based on bagging approach to generate a group of decision trees to construct a more reliable model by minimizing residual values. The RF produces a multiple regression trees among training data set by applying bootstrap technique^76–78^. The final result can be obtained using averaging process of random vectors ($\Theta$). In this stage, the correlation between regression trees is reduced, leading to the reduction of variance value^79–81^. For each tree predictor *h*(*X*,$\Theta$), mean-squared generalization error values can be calculated by using numerical estimator *h*(*X*), which can be expressed as follows:

11 $$E_{X,Y} (Y - h(X))^{2}$$

If there is a large number of decision trees during averaging process, two theorems should be considered as follows:

#### ***Theorem 1***

By increasing the number of trees, the generalization error of the forest can be calculated as follows:

12 $$E_{X,Y} (Yav_{j} - h(X\Theta ))^{2} \to E_{X,Y} (Y - E_{\Theta } h(X,\Theta ))^{2}$$

The average generalization error (GE) can be expressed as follows:

13 $$PE*(tree) = E_{\Theta } E_{X,Y} (Y - E_{\Theta } h(X,\Theta ))^{2}$$

In fact, this theorem shows that over-fitting problems will not take place by increasing number of trees in the RF model and it tries to keep generalization performance well using a GE mentioned earlier.

#### ***Theorem 2***

By considering $EY = E_{X} h(X,\Theta )$, the upper bound for the GE can be computed as follows:

14 $$PE*(forest) \le \overline{\rho }.PE*(tree)$$

where $\overline{\rho }$ denotes the weighted correlation. More detail of the RF can be found in Breiman^75^.

### Artificial neural network-multilayer perceptron (ANNMLP)

**Figure 3:**
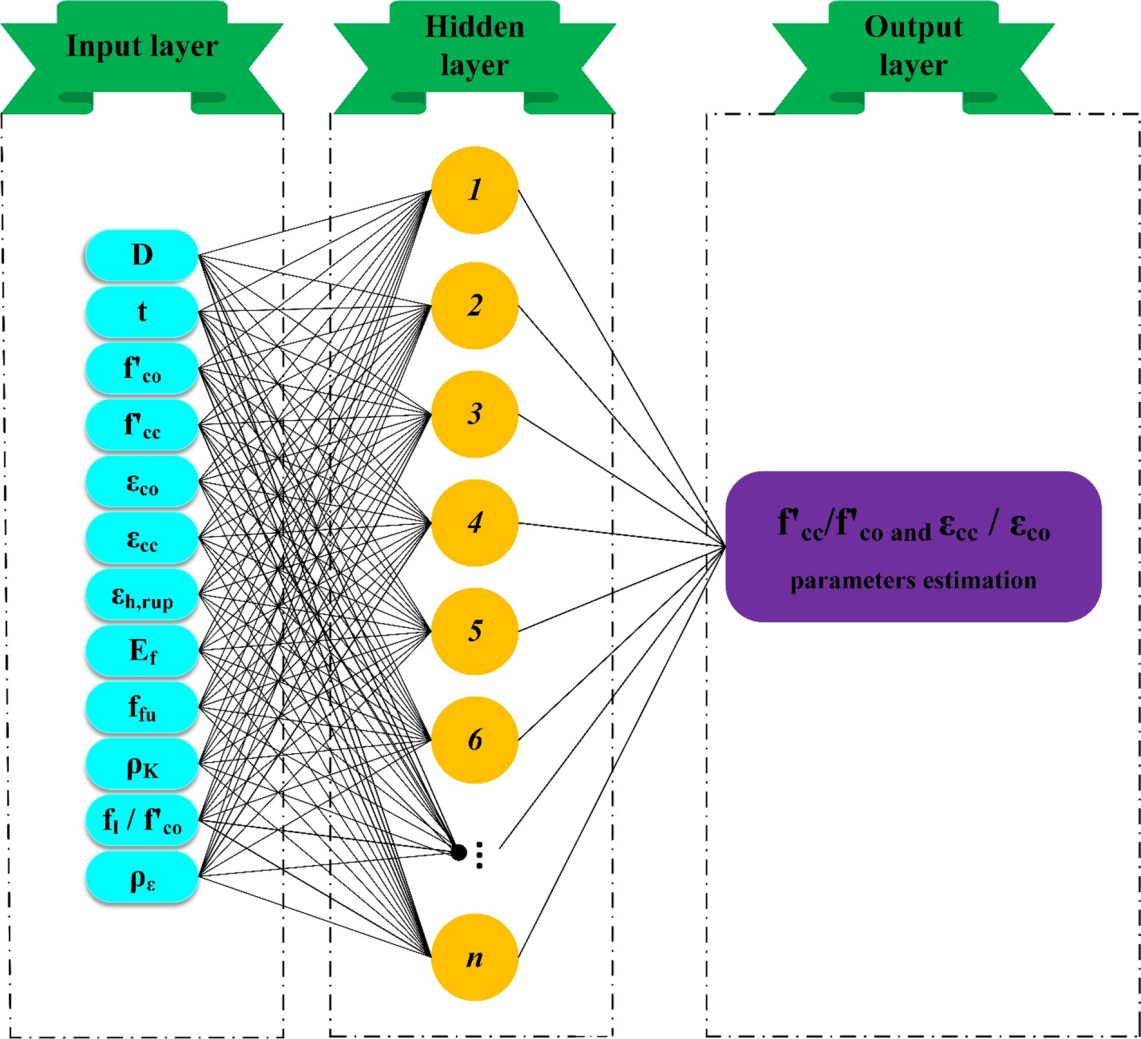
Schematic architecture of ANNMLP.

The MLP as the most typical ANN is a robust mathematical model similar to human brain and can be represented as a biological system. This enables the model to extract input and output parameters relationship by using elements called neurons in each layer^82–84^. The general mathematical formulation of the ANNMLP can be represented as follows^85^:

15 $$O(x) = f\left( {w_{O} + \sum\limits_{j = 1}^{J} {W_{j} .f\left( {W_{Oj} + W_{j}^{T} x} \right)} } \right)$$

where x, W_Oj_, and W_O_ are input vector, hidden layer weight vector and vector of weights in the output layer, respectively. W_j_ and f indicate the synaptic weight and activation function, respectively. As mentioned before, the MLP consists of multiple layers, such as the input layer (IL), hidden layer (HL), and output layer (OL)^86–88^. Receiving input and output parameters is the task of the IL. In the HL, neurons by extracting patterns and relationships between input and output parameters compute weights and biases using optimization process to reduce the difference between observed and modelled values^89^. Finally, in the OL final results are computed. More detail of the ANNMLP model can be found in^90–92^. Figure 3 shows schematic structure of ANNMLP. In this study, a ML model based on the ANNMLP with a single HL was applied. In addition, Bayesian Regulation optimization technique was utilized to train the model. It should be mentioned that sigmoid activation function was utilized in the hidden layer (HL), while the output layer (OL) employed the linear activation function. The formulation of sigmoid function can be presented as follows:

16 $$f(x) = \frac{2}{{1 + e^{ - 2x} }} - 1$$

### Artificial neural network-radial basis function (ANNRBF)

The RBF is another branch of the ANN with a reliable performance in describing highly complex problems^93^^,^^94^^,^^91^. The only difference between the ANNMLP and ANNRBF is that the ANNRBF has one HL. The HL of the ANNRBF consists of several nodes and biases. In the training phase of the ANNRBF, input signals are transferred from IL to HL. By considering the spread coefficient and the center, the position of the input vector compared to the center is computed accordingly. The Euclidian norm is represented as follows:

17 $$r_{i} = \sqrt {\sum\limits_{k = 1}^{d} {(x_{k} - c_{ki} )^{2} } }$$

where x, c_ik_ and d denote input vector, center and number of data samples, respectively. Gaussian function is one of the most applicable RBFs, which can be defined as below:

18 $$\phi (r)=\mathit{exp}\left[\frac{{r}^{2}}{2{\omega }^{2}}\right]$$

in which r, $\omega$ and $\phi$ are the Euclidian distance, the Gaussian function and the spread coefficient, respectively. The general mathematical structure of the ANNRBF can be shown as follows^95^^,^^96^:

19 $$Y_{j} = \sum\limits_{i = 1}^{{h_{n} }} {w_{ji} } \phi_{i} (r) + b_{i} ,\begin{array}{*{20}c} {} & {i = 1,...,h_{n} } \\ \end{array} \begin{array}{*{20}c} {} & {and\begin{array}{*{20}c} {} & {j = 1,...,N} \\ \end{array} } \\ \end{array}$$

where b_i_ and w_ji_ denote bias and weight vectors, respectively.

## Quantitative performance metrics

In this study, the predictive performance of models for estimating the ultimate condition of FRP-confined concrete was analyzed through comparisons across multiple statistical measures, specifically the root mean square error (RMSE), coefficient of determination (R^2^), and mean absolute error (MAE), as detailed in the following equations.

20 $$\text{RMSE}=\sqrt{\frac{{\sum }_{i=1}^{n}{((UC{)}_{io}-(UC{)}_{ip})}^{2}}{n}}$$

21 $$\text{MAE}=\frac{1}{N}{\sum }_{i=1}^{N}\left|(UC{)}_{io}-(UC{)}_{ip}\right|$$

22 $${\text{R}}^{2}={\left\{\frac{{\sum }_{i=1}^{n}((UC{)}_{io}-(\overline{UC }{)}_{io})((UC{)}_{ip}-(\overline{UC}{)}_{ip})}{\sqrt{{\sum }_{i=1}^{n}((UC{)}_{io}-\overline{(UC{)}_{io}}{)}^{2}{\sum }_{i=1}^{n}((UC{)}_{ip}-\overline{(UC{)}_{ip}}{)}^{2}}}\right\}}^{2}$$

in which *n* represents the number of data points. $(UC{)}_{io}$ and $(UC{)}_{ip}$ symbolize the observed and predicted values, respectively, for parameters defining the ultimate condition of FRP-confined concrete. These parameters are the compressive strength and ultimate axial strain of concrete composite cores reinforced with FRP jacket.

## Results and discussion

The design of FRP-confined concretes requires the precise assessment of their performance due to the confinement provided by FRP composites, which enhances strength and strain of concrete core^9,13, 97, 98^. In this study, the prediction issue for strength (*f′*_*cc*_*/f′*_*co*_) and strain (*ε*_*cc*_/*ε*_*co*_) ratios of confinement of FRP-confined concrete was accomplished utilizing diverse ML (i.e., GBRT, RF, ANNMLP, and ANNRBF) and empirical (i.e., Keshtegar et al.^6,7^, Ozbakkaloglu and Lim^10^, Sadeghian and Fam^12^, Pham and Hadi^11^, and Wu and Wei^15^) models. Table 1 presents statistical properties of variables employed in this study. The variables consist of concrete diameter (*D*), total FRP thickness (*t*), unconfined concrete strength (*f′*_*co*_), compressive strength of confined concrete (*f′*_*cc*_), axial strain corresponding to the unconfined concrete strength (*ε*_*co*_), ultimate axial strain of confined concrete (*ε*_*cc*_), hoop rupture strain of FRP (*ε*_*h,rup*_), elastic modulus of fiber (*E*_*f*_), tensile strength of fiber (*f*_*fu*_), confinement stiffness ratio (*ρ*_*K*_), lateral confining pressure ratio (*f*_*l*_*/f′*_*co*_), strain ratio (*ρ*_*ε*_), lateral confining pressure (*f*_*l*_), strength ratio of confinement (*f′*_*cc*_*/f′*_*co*_), and strain ratio of confinement (*ε*_*cc*_/*ε*_*co*_). Based on the Table 1, the *f*_*fu*_ provided the highest standard deviation, which was far from the mean *f*_*fu*_ among all variables. In addition, the *f*_*fu*_ showed the only negative skewness among all variables.

### Predicting strength ratio of confinement (*f′*_*cc*_*/f′*_*co*_) of FRP-confined concrete

#### Machine learning models

The performance of different ML models for predicting *f′*_*cc*_*/f′*_*co*_ of FRP-confined concrete based on three evaluation measures of RMSE, MAE, and R^2^ is displayed in Table 4. As can be seen, the predicted values of *f′*_*cc*_*/f′*_*co*_ utilizing GBRT (RMSE = 0.100, MAE = 0.075, and R^2^ = 0.985) were better than those of the RF, ANNMLP, and ANNRBF during training phase. In addition, the GBRT (RMSE = 0.153, MAE = 0.104, and R^2^ = 0.948) was superior than the other ML models during testing phase.

**Table 4:** Model performance for predicting strength ratio of confinement (*f′*_*cc*_*/f′*_*co*_).

Models	Training			Testing		
	RMSE	MAE	R^2^	RMSE	MAE	R^2^
*Soft computing models*						
GBRT	**0.100**	**0.075**	**0.985**	**0.153**	**0.104**	**0.948**
RF	0.118	0.063	0.982	0.185	0.113	0.932
ANNMLP	0.220	0.144	0.930	0.154	0.118	0.947
ANNRBF	0.117	0.072	0.979	0.461	0.217	0.580
*Empirical models*						
Keshtegar et al.^6,7^	0.356	0.248	0.812	0.284	0.206	0.823
Ozbakkaloglu and Lim^10^	0.405	0.261	0.765	0.285	0.205	0.826
Sadeghian and Fam^12^	0.373	0.249	0.799	0.288	0.207	0.818
Pham and Hadi^11^	0.588	0.433	0.770	0.509	0.386	0.811

The values in bold indicate that the model is optimal.

**Figure 4:**
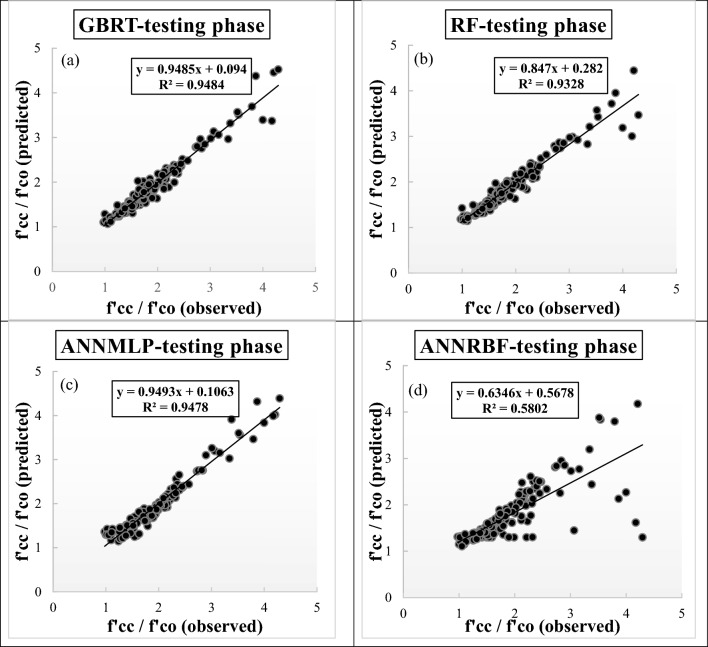
(**a**)**–**(**d**) Scatter plots for predicting strength ratio of confinement (*f′*_*cc*_*/f′*_*co*_) utilizing different soft computing models during testing phase, (**a**) GBRT, (**b**) RF, (**c**) ANNMLP, and (**d**) ANNRBF.

Figure 4a–d present the scatter plots of observed and predicted values of *f′*_*cc*_*/f′*_*co*_ employing different ML models during testing phase. The R^2^ value, regression equation, and optimized line for corresponding individual diagram are embedded in the specific diagrams. It can be inferred from Figs. 4a–d that an obvious difference existed between the individual ML model. The GBRT provided the maximum R^2^ value (0.9484) compared to other ML models during testing phase, while the ANNRBF had the worst R^2^ value (0.5802) during testing phase.

**Figure 5:**
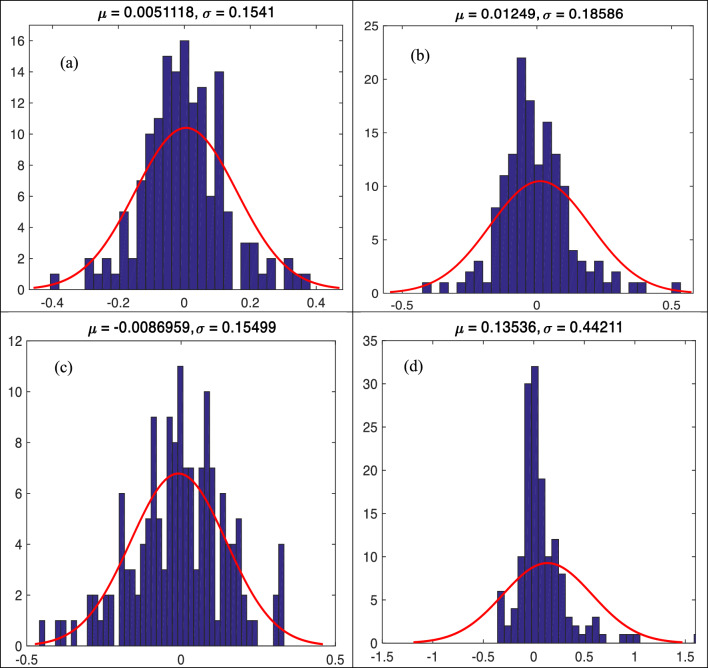
(**a**)**–**(**d**) Error histogram for predicting strength ratio of confinement (*f′*_*cc*_*/f′*_*co*_) utilizing different soft computing models during testing phase, (**a**) GBRT, (**b**) RF, (**c**) ANNMLP, and (**d**) ANNRBF.

Figure 5a–d show the error histogram of *f′*_*cc*_*/f′*_*co*_ including mean (μ) and standard deviation (σ) of predicted error values utilizing different ML models during testing phase. They show that the GBRT and ANNMLP had the lowest σ (0.15410 and 0.15499, respectively), while the ANNRBF had the highest σ (0.44211). This arrangement follows the performance of RMSE values between the observed and predicted *f′*_*cc*_*/f′*_*co*_ utilizing different ML models during testing phase.

#### Empirical models

The performance of different empirical models for predicting *f′*_*cc*_*/f′*_*co*_ of FRP-confined concrete based on three evaluation measures of RMSE, MAE, and R^2^ is presented in Table 4. It can be observed from the table that the predicted values of *f′*_*cc*_*/f′*_*co*_ proposed by Keshtegar et al.^6,7^ (RMSE = 0.356, MAE = 0.248, and R^2^ = 0.812) were superior to those of Ozbakkaloglu and Lim^10^^,^ Sadeghian and Fam^12^, and Pham and Hadi^11^ during training phase. In addition^6,6^, (RMSE = 0.284, MAE = 0.206, and R^2^ = 0.823) and Ozbakkaloglu and Lim^10^ (RMSE = 0.285, MAE = 0.205, and R^2^ = 0.826) provided more accurate predictions than the other empirical models during testing phase.

**Figure 6:**
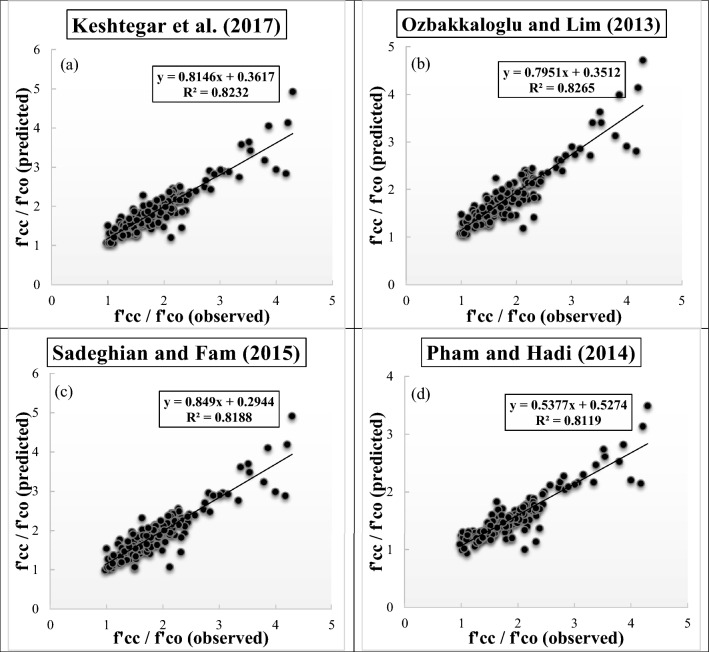
(**a**)**–**(**d**) Scatter plots for predicting strength ratio of confinement (*f′*_*cc*_*/f′*_*co*_) utilizing diverse empirical models during testing phase, (**a**) Keshtegar et al.^6,7^, (**b**) Ozbakkaloglu and Lim^10^, (**c**) Sadeghian and Fam^12^, (**d**) Pham and Hadi^11^.

Figure 6a–d present the scatter plots of observed and predicted values of *f′*_*cc*_*/f′*_*co*_ utilizing diverse empirical models during testing phase. The R^2^ value, regression equation, and fitted line for corresponding each figure are enclosed in the figures. It is observed from Fig. 6a–d that a clear disparity can be identified from the corresponding empirical models. Ozbakkaloglu and Lim^10^ provided the highest R^2^ value (0.8265) during testing phase compared to the other empirical models, while Pham and Hadi^11^ exhibited the worst R^2^ value (0.8119) during testing phase.

**Figure 7:**
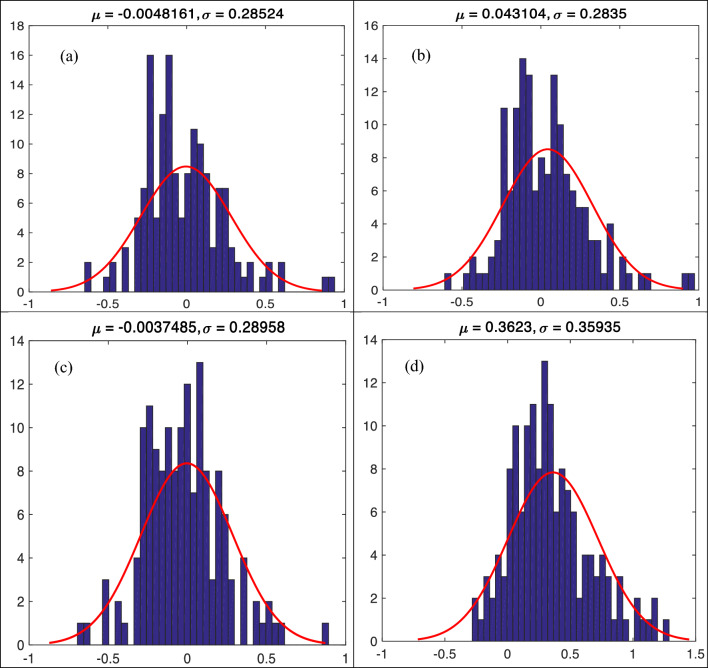
(**a**)**–**(**d**) Error histogram for predicting strength ratio of confinement (*f′*_*cc*_*/f′*_*co*_) utilizing diverse empirical models during testing phase, (**a**) Keshtegar et al.^6,7^, (**b**) Ozbakkaloglu and Lim^10^, (**c**) Sadeghian and Fam^12^, (**d**) Pham and Hadi^11^.

Figure 7a–d display the error histogram of *f′*_*cc*_*/f′*_*co*_ including μ and σ of predicted error values utilizing diverse empirical models during testing phase. According to the figures, Keshtegar et al. ^6,7^and Ozbakkaloglu and Lim^10^ supplied the lowest σ (0.28524 and 0.28350), while Pham and Hadi^11^ yielded the highest σ (0.35935). This trend trails the behavior of RMSE values between observed and predicted *f′*_*cc*_*/f′*_*co*_ utilizing diverse empirical models during testing phase.

### Predicting strain ratio of confinement (*ε*_*cc*_/*ε*_*co*_) of FRP-confined concrete

#### Machine learning models

The performance of different ML models for predicting *ε*_*cc*_/*ε*_*co*_ of FRP-confined concrete based on RMSE, MAE, and R^2^ is shown in Table 5. According to the table, the GBRT (RMSE = 0.801, MAE = 0.605, and R^2^ = 0.995) had more accurate predictions than the RF, ANNMLP, and ANNRBF during training phase. Further, the GBRT (RMSE = 0.765, MAE = 0.592, and R^2^ = 0.995) and ANNMLP (RMSE = 0.691, MAE = 0.539, and R^2^ = 0.976) were superior to the RF and ANNRBF during testing phase.

**Table 5:** Model performance for predicting strain ratio of confinement (*ε*_*cc*_/*ε*_*co*_).

Models	Training			Testing		
	RMSE	MAE	R^2^	RMSE	MAE	R^2^
*Soft computing models*						
GBRT	0.801	0.605	0.995	0.765	0.592	0.995
RF	0.537	0.327	0.989	0.779	0.542	0.972
ANNMLP	**0.981**	**0.560**	**0.963**	**0.691**	**0.539**	**0.976**
ANNRBF	0.745	0.490	0.975	2.777	1.612	0.692
*Empirical models*						
Keshtegar et al.^6,7^	2.626	1.868	0.690	2.217	1.611	0.758
Ozbakkaloglu and Lim^10^	3.197	2.128	0.619	2.622	1.860	0.696
Sadeghian and Fam^12^	2.634	1.864	0.689	2.265	1.633	0.755
Wu and Wie^15^	4.255	3.266	0.628	4.250	3.340	0.697

The values in bold indicate that the model is optimal.

**Figure 8:**
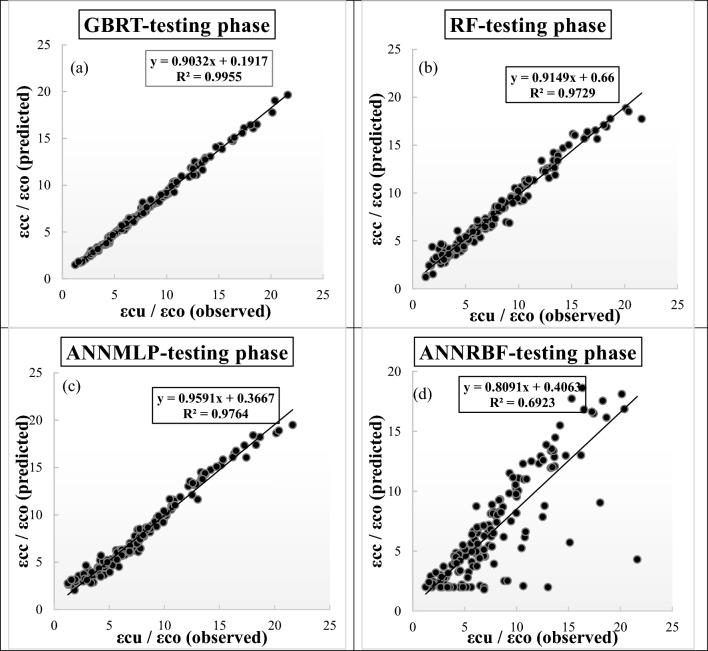
(**a**)–(**d**) Scatter plots for predicting strain ratio of confinement (*ε*_*cc*_/*ε*_*co*_) utilizing different soft computing models during testing phase, (**a**) GBRT, (**b**) RF, (**c**) ANNMLP, and (**d**) ANNRBF.

Figure 8a–d illustrate the scatter plots of observed and predicted values of *ε*_*cc*_/*ε*_*co*_ utilizing different ML models during testing phase. The R^2^ value, regression equation, and optimized line are inserted in each figure. It can be seen from Fig. 8a–d that there was an obvious difference between each ML model. The GBRT provided the highest R^2^ value (0.9955) and the ANNRBF exhibited the lowest R^2^ value (0.6923) during testing phase.

**Figure 9:**
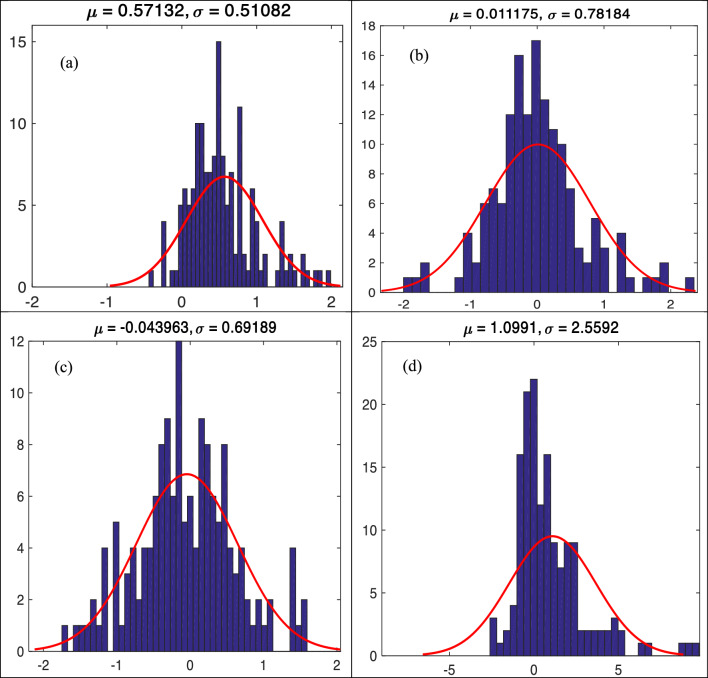
(**a**)–(**d**) Error histogram for predicting strain ratio of confinement (*ε*_*cc*_/*ε*_*co*_) utilizing different soft computing models during testing phase, (**a**) GBRT, (**b**) RF, (**c**) ANNMLP, and (**d**) ANNRBF.

Figure 9a–d define the error histogram of *ε*_*cc*_/*ε*_*co*_ including μ and σ of predicted error values utilizing different ML models during testing phase. Based on the figure, the GBRT yielded the lowest σ (0.51082), whereas the ANNRBF yielded the highest σ (2.55920). This arrangement follows the appearance of RMSE values between the observed and predicted *ε*_*cc*_/*ε*_*co*_ utilizing different ML models during testing phase.

#### Empirical models

Table 5 presents the performance of different empirical models for predicting *ε*_*cc*_/*ε*_*co*_ of FRP-confined concrete based on different metrics. It can be seen from the table that the predictions of *ε*_*cc*_/*ε*_*co*_ by Keshtegar et al.^52,53^ (RMSE = 2.626, MAE = 1.868, and R^2^ = 0.690) were more outstanding than those of Ozbakkaloglu and Lim^10^, Sadeghian and Fam^12^, and Wu and Wei^15^ during training phase. In addition, Keshtegar et al.^6,7^ (RMSE = 2.217, MAE = 1.611, and R^2^ = 0.758) supplied more accurate predictions than other empirical models during testing phase.

**Figure 10:**
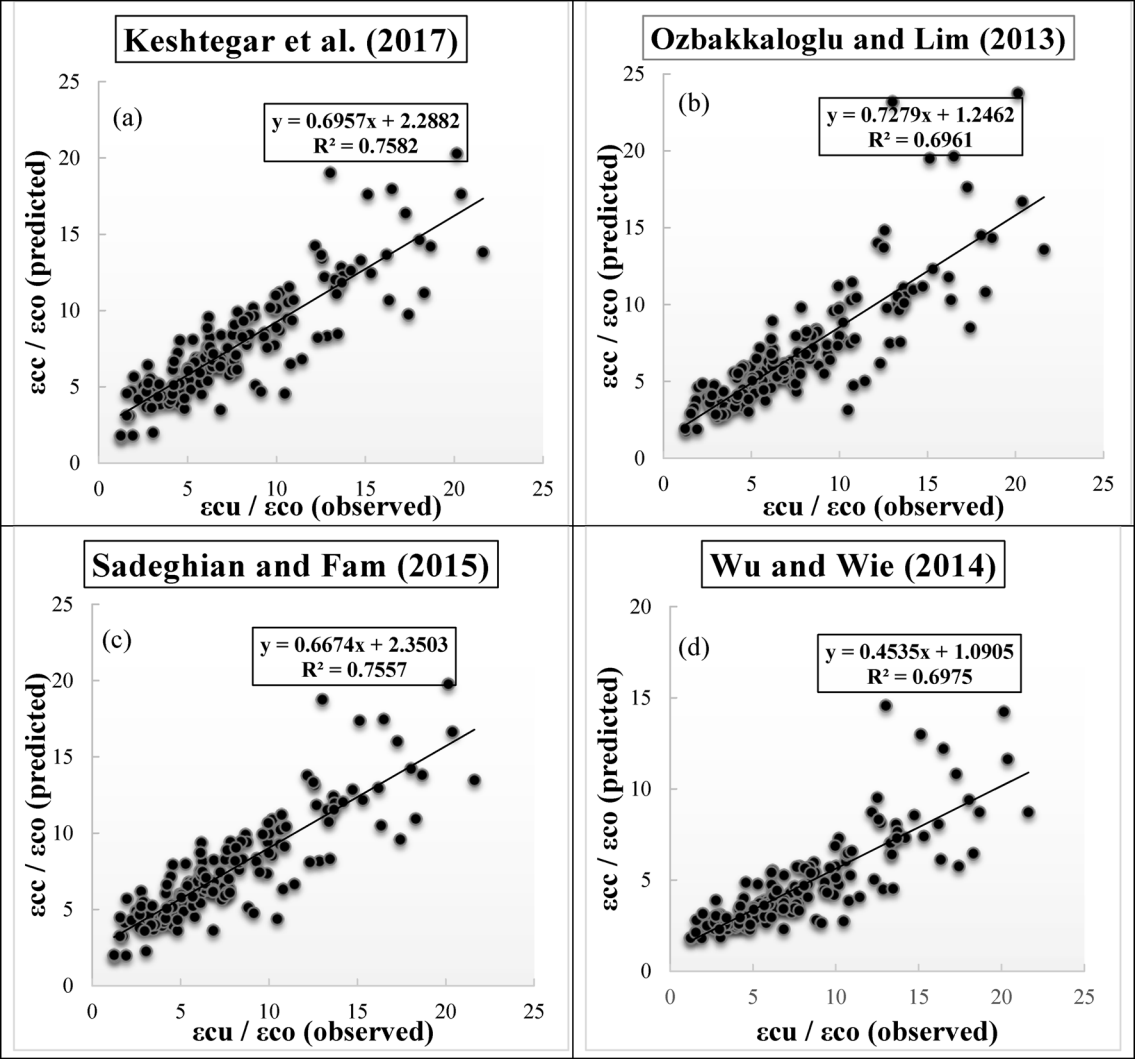
(**a**)–(**d**) Scatter plots for predicting strain ratio of confinement (*ε*_*cc*_/*ε*_*co*_) utilizing diverse empirical models during testing phase, (**a**) Keshtegar et al.^6,7^, (**b**) Ozbakkaloglu and Lim^10^, (**c**) Sadeghian and Fam^12^, (**d**) Wu and Wie^15^.

Figure 10a–d show the scatter plots of observed and predicted values of *ε*_*cc*_/*ε*_*co*_ utilizing diverse empirical models during testing phase, including the R^2^ value, regression equation, and fitted line. A distinct discrepancy can be recognized from the empirical models in the figures. Keshtegar et al.^6,7^ yielded the highest R^2^ value (0.7582) and Wu and Wei^15^ supplied the lowest R^2^ value (0.6975) during testing phase.

**Figure 11:**
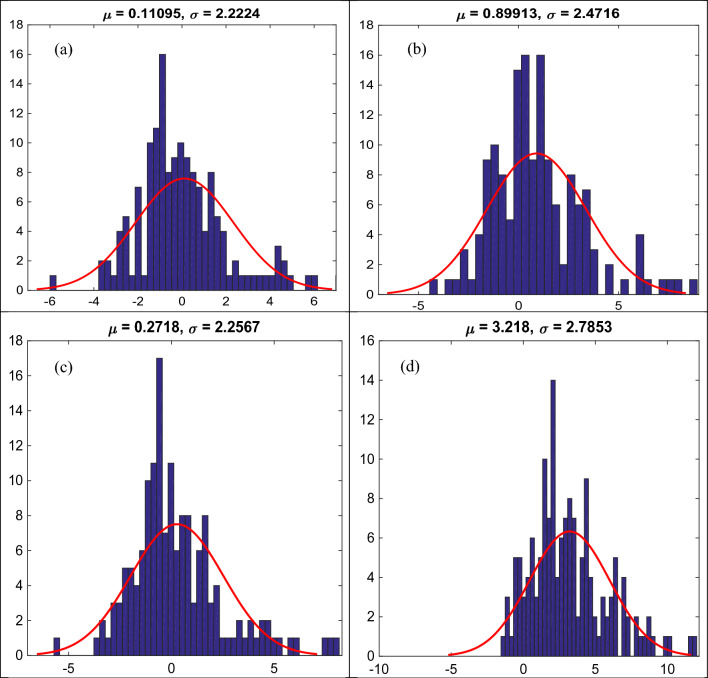
(**a**)–(**d**) Error histogram for predicting strain ratio of confinement (*ε*_*cc*_/*ε*_*co*_) utilizing diverse empirical models during testing phase, (**a**) Keshtegar et al.^6,7^, (**b**) Ozbakkaloglu and Lim^10^, (**c**) Sadeghian and Fam^12^, (**d**) Wu and Wie^15^.

Figure 11a–d show the error histogram of *ε*_*cc*_/*ε*_*co*_ including μ and σ of predicted error values utilizing diverse empirical models during testing phase. As shown, Keshtegar et al.^6,7^ supplied the lowest σ (2.2224), whereas Wu and Wei^15^ yielded the highest σ (2.7853). This observation trails the behavior of RMSE values between observed and predicted *ε*_*cc*_/*ε*_*co*_ utilizing diverse empirical models during testing phase.

### Visual assistance for predicting strength (*f′*_*cc*_*/f′*_*co*_) and strain (*ε*_*cc*_/*ε*_*co*_) ratio of confinement of FRP-confined concrete

**Figure 12:**
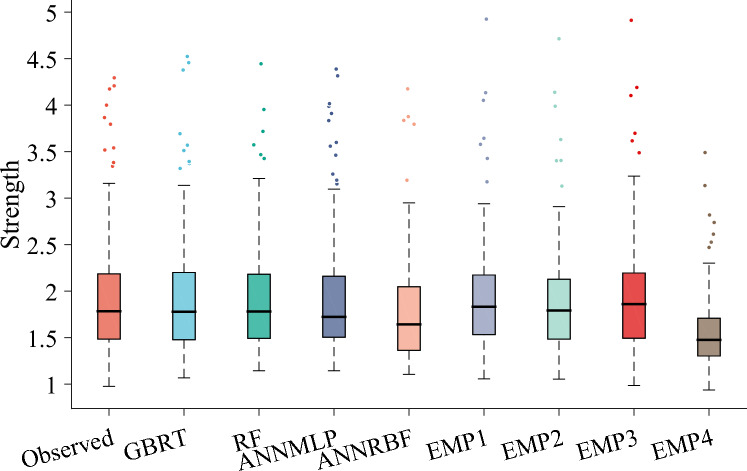
Boxplots for predicting strength ratio of confinement (*f′*_*cc*_*/f′*_*co*_) utilizing different soft computing and empirical models during testing phase, EMP1, EMP2, EMP3, and EMP4 denotes Keshtegar et al.^6,7^, Ozbakkaloglu and Lim^10^, Sadeghian and Fam^12^, and Pham and Hadi^11^, respectively.

Two visual tools including Boxplots^99^ and Taylor diagram^100^ were applied to validate the performances of the ML and empirical models. Figure 12 presents the boxplots for prediction of *f′*_*cc*_*/f′*_*co*_ employing different models during testing phase. It can be seen that the GBRT resembled the parameters of boxplot including shape (lowest and highest values, first and third quartile, and median) and length (lowest and highest points) of observed boxplot compared to the other ML (i.e., RF, ANNMLP, and ANNRBF) and all the empirical (i.e., Keshtegar et al.^6,7^, Ozbakkaloglu and Lim^10^, Sadeghian and Fam^12^, and Pham and Hadi^11^ models. In addition, the ANNMLP slightly featured the characteristics (i.e., shape and length) of observed boxplot compared to the RF, ANNRBF, and all the empirical models.

**Figure 13:**
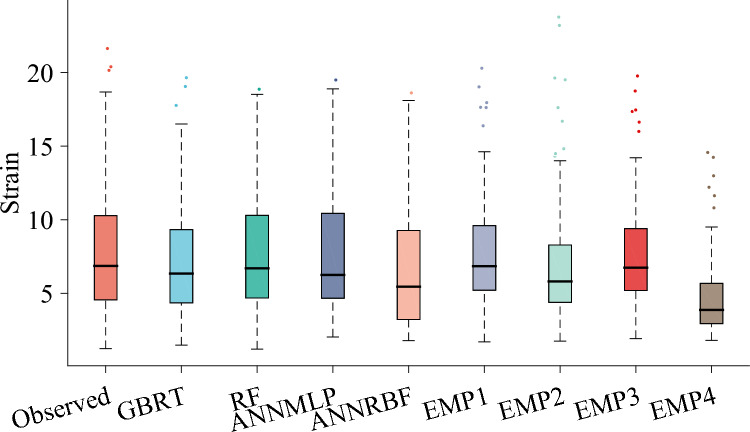
Boxplots for predicting strain ratio of confinement (*ε*_*cc*_/*ε*_*co*_) utilizing different soft computing and empirical models during testing phase, EMP1, EMP2, EMP3, and EMP4 denotes Keshtegar et al.^6,7^, Ozbakkaloglu and Lim^10^, Sadeghian and Fam^12^, and Pham and Hadi^11^ or Wu and Wie^15^, respectively.

Figure 13 illustrates the boxplots for prediction of *ε*_*cc*_/*ε*_*co*_ utilizing different models during testing phase. It can be seen that the RF coincided the parameters of boxplot such as shape and length of observed boxplot compared to the other ML and all the empirical models. Furthermore, the ANNMLP marginally duplicated the characteristics (i.e., shape and length) of the observed boxplot compared to the GBRT, ANNRBF, and all the empirical models.

**Figure 14:**
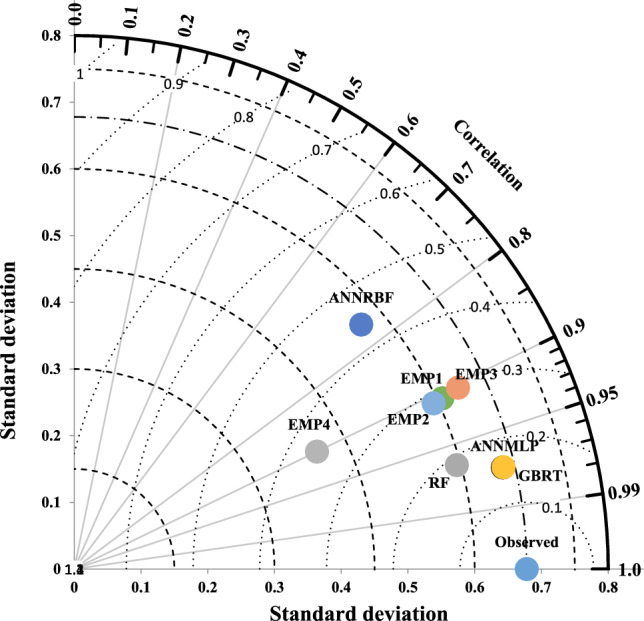
Taylor diagram for predicting strength ratio of confinement (*f′*_*cc*_*/f′*_*co*_) utilizing different soft computing and empirical models during testing phase, EMP1, EMP2, EMP3, and EMP4 denote^6,6^, Ozbakkaloglu and Lim^10^, Sadeghian and Fam^12^, and Pham and Hadi^11^, respectively.

**Figure 15:**
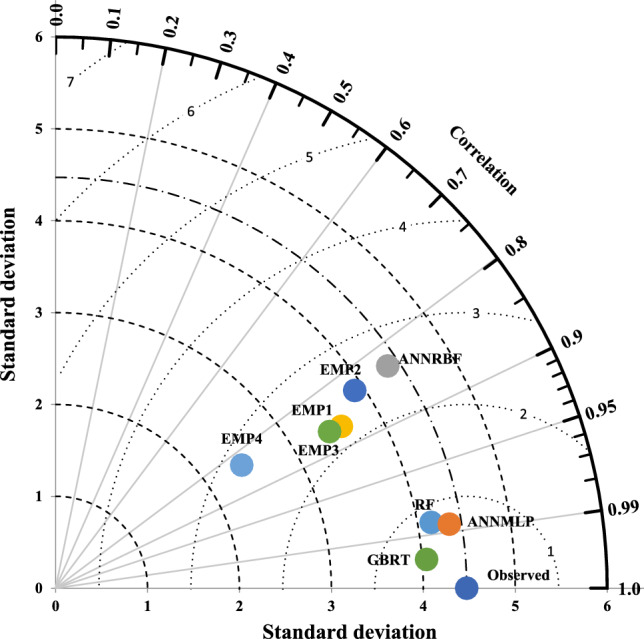
Taylor diagram for predicting strain ratio of confinement (*ε*_*cc*_/*ε*_*co*_) utilizing different soft computing and empirical models during testing phase, EMP1, EMP2, EMP3, and EMP4 denote^6,6^, Ozbakkaloglu and Lim^10^, Sadeghian and Fam^12^, and Wu and Wie^15^, respectively.

Taylor diagram, shown in Figs. 14 and 15, implements three statistical measures including normalized standard deviation (NSD), correlation coefficient (CC), and RMSE for drawing of diagram’s structure. The employment of the Taylor diagram can notice the precise model with the predicted strength (Fig. 14) and strain (Fig. 15) ratio of confinement with polar axis (NSD) and radial axis (CC). As can be seen in Fig. 14, since the points of the GBRT and ANNMLP had the shortest distances from those of observed *f′*_*cc*_*/f′*_*co*_, the GBRT and ANNMLP were the best accurate models for predicting *f′*_*cc*_*/f′*_*co*_ among all other models. On the other hand, because the point of the ANNRBF had the longest path from the observed point, the ANNRBF was the worst precise model for predicting *f′*_*cc*_*/f′*_*co*_ during testing phase. Based on Fig. 15, the node of the GBRT was the nearest to the observed *ε*_*cc*_/*ε*_*co*_, whereas the node of the ANNRBF, Ozbakkaloglu and Lim^10^^,^ and Wu and Wei^15^ had the longest distance from the observed *ε*_*cc*_/*ε*_*co*_. Therefore, Taylor diagram displayed more predictive efficiency for performance of the GBRT compared to the other ML and all the empirical models during testing phase.

### Discussion

The present study assessed the predictive ability of different machine learning (i.e., GBRT, RF, ANNMLP, and ANNRBF) and empirical models (i.e., Keshtegar et al.^6,7^, Ozbakkaloglu and Lim^10^, Sadeghian and Fam^12^, Pham and Hadi^11^, and Wu and Wei^15^ for strength ratio of confinement (*f′*_*cc*_*/f′*_*co*_) and strain ratio of confinement (*ε*_*cc*_/*ε*_*co*_) of FRP-confined concrete. Based on implementing three evaluation measures and visual assistances, the GBRT provided the best accuracy for predicting *f'*_*cc*_*/f'*_*co*_ and *ε*_*cc*_/*ε*_*co*_ among machine learning and empirical models during training and testing phases. In addition, the comparison between machine learning and empirical models provided that the GBRT, RF, and ANNMLP were superior to all the empirical models for predicting *f′*_*cc*_*/f′*_*co*_ and *ε*_*cc*_/*ε*_*co*_ during training and testing phases. The ANNRBF provided accurate prediction of *f′*_*cc*_*/f′*_*co*_ and *ε*_*cc*_/*ε*_*co*_ during training phase, while it gave the least accurate prediction of *f′*_*cc*_*/f′*_*co*_ and *ε*_*cc*_/*ε*_*co*_ during testing phase compared to other machine learning and empirical models. In addition, Wu and Wei^15^ provided the similar predictive results to the ANNRBF during testing phase. In addition, the comparison among all empirical models indicated that^6,7^ suggested accurate prediction of *f′*_*cc*_*/f′*_*co*_ and *ε*_*cc*_/*ε*_*co*_ compared to other empirical models during training phase. However, the values of *f′*_*cc*_*/f′*_*co*_ by Ozbakkaloglu and Lim^10^ and *ε*_*cc*_/*ε*_*co*_ by Keshtegar et al.^6,7^ provided accurate prediction compared to other empirical models during testing phase, respectively. In this study, Keshtegar et al.^6,7^ suggested accurate performance for predicting the values of *f′*_*cc*_*/f′*_*co*_ and *ε*_*cc*_/*ε*_*co*_ compared to other empirical models. To achieve the reliable and accurate solution for predicting the values of *f′*_*cc*_*/f′*_*co*_ and *ε*_*cc*_/*ε*_*co*_, however, diverse dataset in the research field have to be applied.

Granting the leading model conditional on individual R^2^ values, the GBRT, which supplied the best prediction, boosted the predictive efficiency of *f′*_*cc*_*/f′*_*co*_ for machine learning and empirical models by 1.72% (RF), 0.11% (ANNMLP), 63.45% (ANNRBF), 15.19% Keshtegar et al.^6,7^, 14.77% Ozbakkaloglu and Lim^10^, 15.89% Sadeghian and Fam^12^, and 16.89% Pham and Hadi^11^ during testing phase. Regarding the perfect model relying on the specific R^2^ values, the GBRT increased the predictive ability of *ε*_*cc*_/*ε*_*co*_ for machine learning and empirical models by 2.37% (RF), 1.95% (ANNMLP), 43.79% (ANNRBF), 31.27%^6,7^, 42.96% Ozbakkaloglu and Lim^10^, 31.79% Sadeghian and Fam^12^, and 42.75% Wu and Wei^15^ during testing phase.

**Figure 16:**
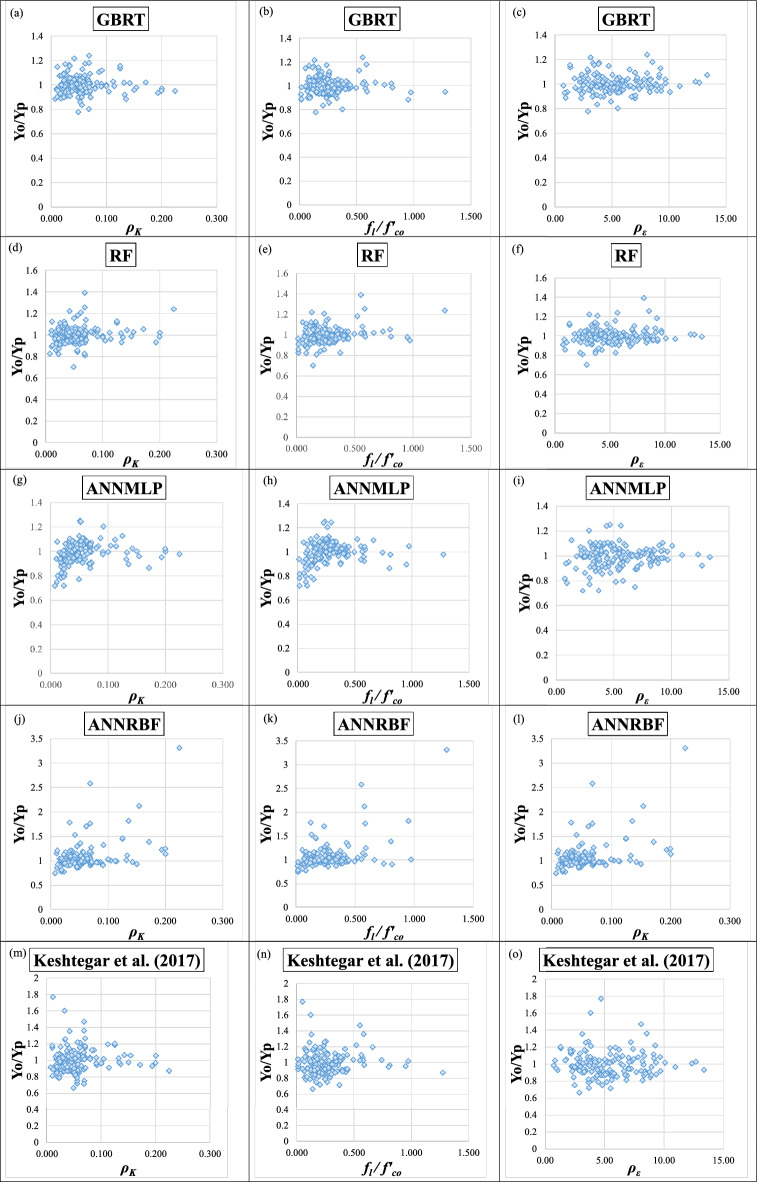
(**a**)–(**o**) The scatter plots for Y_o_/Y_p_ (observed *f′*_*cc*_*/f′*_*co*_ /predicted *f′*_*cc*_*/f′*_*co*_) versus confinement stiffness ratio (*ρ*_*K*_), lateral confining pressure ratio (*f*_*l*_*/f*_*co*_), and strain ratio (*ρ*_*ε*_) utilizing GBRT, RF, ANNMLP, ANNRBF, and empirical model supplied by Keshtegar et al.^6,7^ for predicting strength ratio of confinement (*f*_*cc*_* /f*_*co*_) during testing phase.

**Figure 17:**
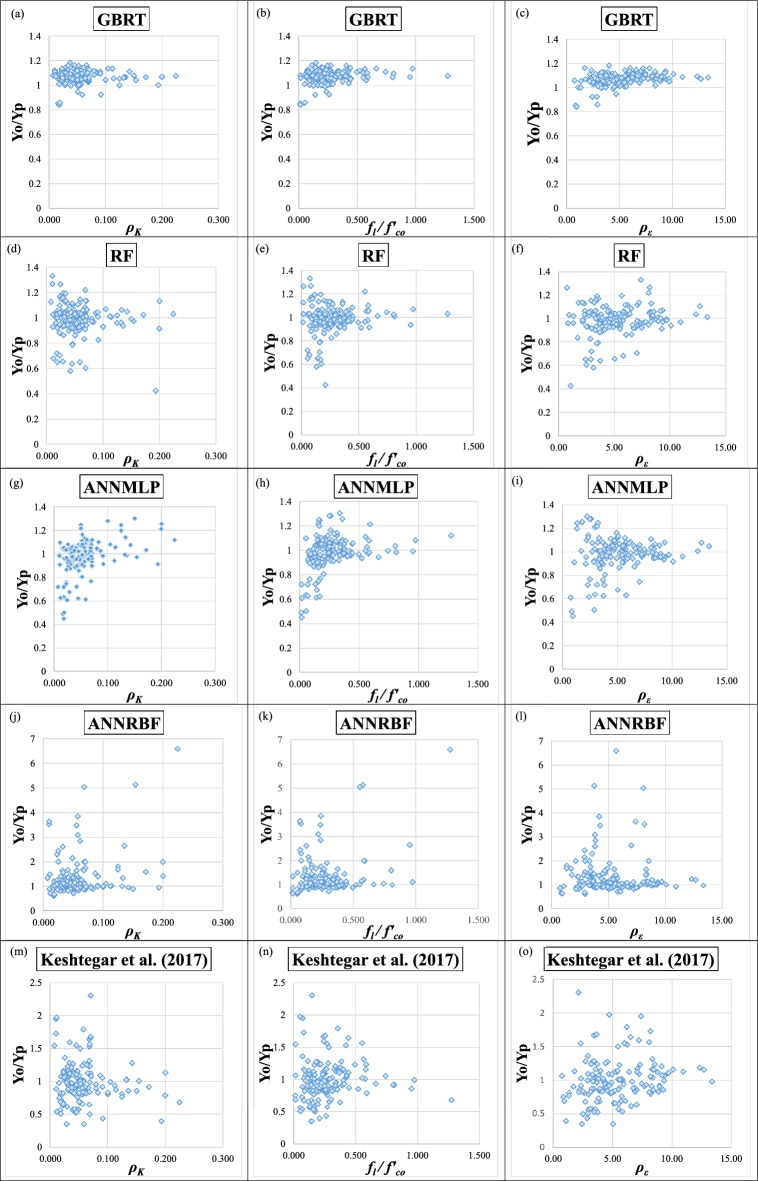
(**a**)–(**o**) The scatter plots for Y_o_/Y_p_ (observed ε_*cc*_/ε_*co*_ /predicted ε_*cc*_/ε_*co*_) versus confinement stiffness ratio (*ρ*_*K*_), lateral confining pressure ratio (*f*_*l*_*/f*_*co*_), and strain ratio (*ρ*_*ε*_) utilizing GBRT, RF, ANNMLP, ANNRBF, and empirical model supplied by Keshtegar et al.^6,7^ during testing phase.

**Figure 18:**
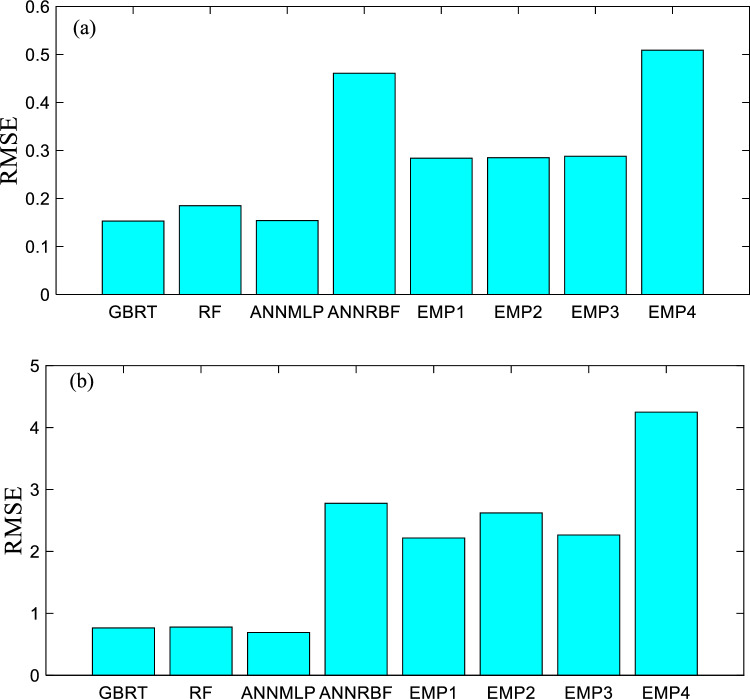
(**a**)–(**b**) Evaluation of the different models in terms of RMSE in testing period for f′cc/f′co and εcc/εco estimation.

**Figure 19:**
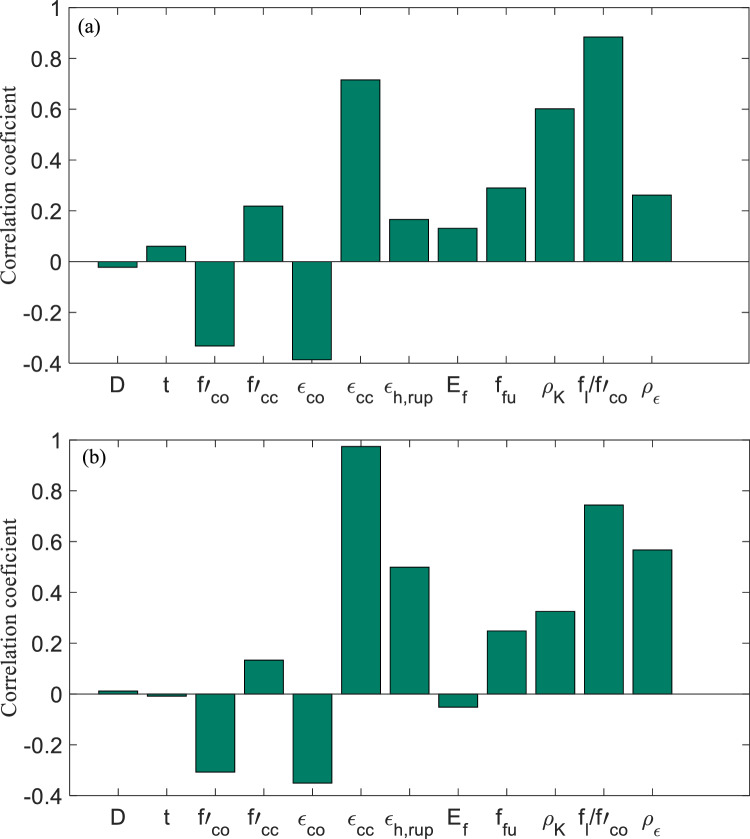
(**a**)–(**b**) Predictor importance analysis using correlation coefficient.

Figures 16a–o and 17a–o present the scatter plots for Y_o_/Y_p_ (i.e., Y_o_ = the ratio between observed *f′*_*cc*_*/f′*_*co*_ and predicted *f′*_*cc*_*/f′*_*co*_ and Y_p_ = the ration between observed ε_*cc*_/ε_*co*_ and predicted ε_*cc*_/ε_*co*_) versus *ρ*_*K*_, *f*_*l*_*/f′*_*co*_, and *ρ*_*ε*_ employing the GBRT, RF, ANNMLP, ANNRBF, and Keshtegar et al.^6,7^ for predicting *f′*_*cc*_*/f′*_*co*_ and *ε*_*cc*_/*ε*_*co*_ during testing phase, respectively. It can be seen that the distribution of data group for the GBRT was not wide but dense spread compared to the RF, ANNMLP, ANNRBF, and Keshtegar et al.^6,7^ during testing phase. However, the ANNRBF and Keshtegar et al.^6,7^ provided widely distributed data group. Therefore, the more accurate ML model of GBRT for predicting *f′*_*cc*_*/f′*_*co*_ and *ε*_*cc*_/*ε*_*co*_ had the concentrated density of data group corresponding to scatter plots for Y_o_/Y_p_ versus *ρ*_*K*_, *f*_*l*_*/f′*_*co*_, and *ρ*_*ε*_ during testing phase, whereas the least accurate models of ANNRBF and Keshtegar et al.^6,7^ showed the wide spread of data group for previously described scatter plots. Figure 18a–b present a comparative analysis of ML methods and empirical approaches. The assessment focuses on *f′*_*cc*_*/f′*_*co*_ and *ε*_*cc*_/*ε*_*co*_ variables prediction accuracy, measured by RMSE, during the model validation stage. As evident from Fig. 18a–b, models GBRT and ANNMLP demonstrated superior performance compared to the other approaches evaluated. Figure 19a–b show the importance of predictors for *f′*_*cc*_*/f′*_*co*_ and *ε*_*cc*_*/ε*_*co*_ estimation using correlation analysis. As can be seen from Figs. 19a–b, parameters *f*_*l*_*/f′*_*co*_, *ε*_*cc*_, and *ρ*_*K*_ are the most influential predictors for the estimation of *f′*_*cc*_*/f′*_*co*_. Moreover, parameters *ε*_*cc*_, *f*_*l*_*/f′*_*co*_, and *ρ*_ε_ can be considered the most effective input factors for *ε*_*cc*_/*ε*_*co*_ estimation.

Considering previous studies for estimating *f′*_*cc*_*/f′*_*co*_ and *ε*_*cc*_/*ε*_*co*_ of FRP-confined concrete utilizing ML and empirical models, Mansouri et al.^101^ developed four ML models for predicting ultimate strength and strain of FRP-confined concrete. They suggested that ANFIS-SC was better than the other ML models for predicting the ultimate strength and strain of FRP-confined concrete. Keshtegar et al.^2^ proposed hybrid response surface method (RSM)—support vector regression (SVR) model to predict the ultimate strength and strain of FRP-confined concrete. They found that the RSM-SVR predicted the ultimate strength and strain of FRP-confined concrete more accurately compared to individual RSM and SVR models and six empirical models. Ilyas et al.^102^ employed gene expression programming (GEP) to predict the strength of circular CFRP-confined concrete. They found that GEP predicted the strength of the confined concrete more accurately compared to ANN, ANFIS, linear regression, and nonlinear regression models. Du et al.^103^ implemented Bayesian optimized XGB (BO-XGBoost) to forecast the strength and strain of FRP-confined concrete. They revealed that BO-XGBoost was better than XGBoost and six empirical models to forecast the strength and strain of FRP-confined concrete.

In the present study, predicting *f′*_*cc*_*/f′*_*co*_ and *ε*_*cc*_/*ε*_*co*_ of FRP-confined concrete was assessed by some machine learning and empirical models. Thus, additional studies are recommended by employing different soft computing (e.g., machine learning and deep learning) and empirical models to augment the diverse problems for predicting *f′*_*cc*_*/f′*_*co*_ and *ε*_*cc*_/*ε*_*co*_ of FRP-confined concrete. In addition, the hybrid models for coupling the evolutionary strategies and data preprocess with soft computing models are suggested to evaluate the potential prediction accuracy of *f′*_*cc*_*/f′*_*co*_ and *ε*_*cc*_/*ε*_*co*_ of FRP-confined concrete.

## Conclusions

The ML models have gained attraction as an essential tool for engineers working to reinforce concrete with FRPs. Since these models can appraise huge volumes of data very efficiently, finding intricate relationships and patterns among different effective elements can impact strength and strain capacity in reinforcement using FRP. These abilities allow the ML tools to make increasingly accurate results to optimize FRP composite designs by structural engineers. Moreover, after integrating with different FRP composites and confinement layouts, these models offer efficient, statistically validated estimates for how concrete structures will perform. Therefore, reliable modeling of the compressive behavior of FRP-strengthened concrete is essential for optimizing structural engineering. This knowledge enables designers to meet safety standards, prevent excessive material use, reduce costs, and minimize environmental footprint. Thus, this study has investigated the feasibility of four different ML models including gradient boosting regression tree (GBRT), random forest (RF), artificial neural network-multilayer perceptron (ANNMLP) and artificial neural network-radial basis function (ANNRBF) in estimating the compressive behavior of the fiber-reinforced polymer (FRP)-confined concrete at ultimate. The findings were held up against several empirical models including Keshtegar et al.^6,7^, Ozbakkaloglu and Lim^10^, Sadeghian and Fam^12^, Wu and Wei^15^ and Pham and Hadi^11^ for predicting strength ratio of confinement (*f′*_*cc*_*/f′*_*co*_) and strain ratio of confinement (*ε*_*cc*_/*ε*_*co*_). The proposed predictive models were established and verified through training on an extensive dataset derived from published literature. Following conclusions can be obtained:

- The GBRT considerably improved the accuracy of machine learning and empirical models for predicting *f′*_*cc*_*/f′*_*co*_, with average improvements in root mean square error (RMSE) of 17.3%, 0.65%, 66.81%, 46.12%, 46.31%, 46.87% and 69.94% compared to RF, ANNMLP, ANNRBF, Keshtegar et al.^6,7^, Ozbakkaloglu and Lim^10^, Sadeghian and Fam^12^ and Pham and Hadi^11^, respectively. The GBRT (RMSE = 0.153) was followed by ANNMLP (RMSE = 0.154) and RF (RMSE = 0.185) in predicting *f′*_*cc*_*/f′*_*co*_. Moreover, Pham and Hadi^11^ produced the worst predictions with RMSE of 0.509.
- For *ε*_*cc*_/*ε*_*co*_ prediction, the ANNMLP model achieved superior accuracy in terms of enhancement in reducing the RMSE as 9.67%, 11.29%, 75.11%, 68.83%, 73.64%, 69.49% and 83.74% compared to GBRT, RF, ANNRBF, Keshtegar et al.^6,7^, Ozbakkaloglu and Lim^10^, Sadeghian and Fam^12^ and Wu and Wei^15^, respectively. The ANNMLP (RMSE = 0.691) was followed by the GBRT (RMSE = 0.765). Also, Wu and Wei^15^ developed the least accurate forecast for *ε*_*cc*_/*ε*_*co*_.
- Among empirical models, Keshtegar et al.^6,7^ exhibited superior performance in predicting *f′*_*cc*_*/f′*_*co*_ of FRP-confined concrete (RMSE = 0.284) followed by Ozbakkaloglu and Lim^10^ (RMSE = 0.285) and Sadeghian and Fam^12^ (RMSE = 0.288). Moreover, when predicting *ε*_*cc*_/*ε*_*co*_ of FRP-confined concrete^6,6^ (RMSE = 2.217) outperformed the other models, followed by Sadeghian and Fam^12^ (RMSE = 2.265) and Ozbakkaloglu and Lim^10^ (RMSE = 2.622)

This study demonstrated the effectiveness of employing two ML algorithms to predict the compressive behavior of FRP-confined concrete at its ultimate state. The evaluation of the models can be expanded by incorporating data from various databases. The findings of this study can bring valuable insights into the comparative performance, robustness, interpretability, and practical implementation aspects of the GBRT and ANNMLP models in predicting the compressive behavior of FRP-confined concrete. The viability of alternative soft computing models, such as Extreme learning machine, CatBoost, and Support Vector Regression, can also be explored for predicting f′_cc_/f′_co_ and ε_cc_/ε_co_ of FRP-confined concrete.

The implementation of the ML models exhibited significant uncertainties, particularly in their tunable components. This imprecision was often amplified by flawed or inaccurate input information, resulting in a compounding effect on the overall error rate. In this regard, finding and tuning hyperparameters can be accomplished using metaheuristic algorithms to reduce the difference between observed measurements and predicted outcomes. Furthermore, implementing a robust feature selection process can significantly mitigate the overfitting problem by selecting the most influential predictors. Moreover, good local performance was observed in most of the ML models, however, in some cases, their ability to generalize was found to be lacking. Also, a significant degree of unpredictability was noted across these systems. In future works, the integration of these techniques with alternative modeling approaches could be explored. Such a combination might be leveraged to enhance predictive accuracy and reduce the inherent uncertainties that were encountered.

## Data Availability

The data that support the findings of this study are available from the corresponding author upon reasonable request.
